# An Optimality Summary: Secret Key Agreement with Physical Unclonable Functions

**DOI:** 10.3390/e23010016

**Published:** 2020-12-24

**Authors:** Onur Günlü, Rafael F. Schaefer

**Affiliations:** Information Theory and Applications Chair, Technische Universität Berlin, 10623 Berlin, Germany; rafael.schaefer@tu-berlin.de

**Keywords:** physical unclonable functions (PUFs), private authentication, secret key generation, information theoretic privacy, code constructions for security

## Abstract

We address security and privacy problems for digital devices and biometrics from an information-theoretic optimality perspective to conduct authentication, message encryption/decryption, identification or secure and private computations by using a secret key. A physical unclonable function (PUF) provides local security to digital devices and this review gives the most relevant summary for information theorists, coding theorists, and signal processing community members who are interested in optimal PUF constructions. Low-complexity signal processing methods are applied to simplify information-theoretic analyses. The best trade-offs between the privacy-leakage, secret-key, and storage rates are discussed. Proposed optimal constructions that jointly design the vector quantizer and error-correction code parameters are listed. These constructions include modern and algebraic codes such as polar codes and convolutional codes, both of which can achieve small block-error probabilities at short block lengths, corresponding to a small number of PUF circuits. Open problems in the PUF literature from signal processing, information theory, coding theory, and hardware complexity perspectives and their combinations are listed to stimulate further advancements in the research on local privacy and security.

## 1. Motivations

Fundamental advances in cryptography were made in secret during the 20th century. One exception was Claude E. Shannon’s paper “Communication Theory of Secrecy Systems” [1]. Until 1967, the literature on security was not extensive, but a book [2] with a historical review of cryptography changed this trend [3]. Since then, the amount of sensitive data to be protected against attackers has increased significantly. Continuous improvements in security are needed and every improvement creates new possibilities for attacks [4].

Recent hardware-intrinsic security systems, biometric secrecy systems, 5th generation of cellular mobile communication networks (5G) and beyond, as well as the internet of things (IoT) networks, have numerous noticeable characteristics that differentiate them from existing mechanisms. These include large numbers of low-complexity terminals with light or no infrastructure, stringent constraints on latency, and primary applications of inference, data gathering, and control. Such characteristics make it difficult to achieve a sufficient level of secrecy and privacy. Traditional cryptographic protocols, requiring certificate management or key distribution, might not be able to handle various applications supported by such technologies and might not be able to assure the privacy of personal information in the data collected. Similarly, low complexity terminals might not have the necessary processing power to handle such protocols, or latency constraints might not permit the processing time required for cryptographic operations. Similarly, traditional methods that store a secret key in a secure nonvolatile memory (NVM) can be illustrated to be not secure because of possible invasive attacks to the hardware. Thus, secrecy and privacy for information systems are issues that need to be rethought in the context of recent networks, digital circuits, and database storage.

Information-theoretic security is an emerging approach to provide secrecy and privacy, for example, for wireless communication systems and networks by exploiting the unique characteristics of the wireless communication channel. Information-theoretic security methods such as physical layer security (PLS) use signal processing, advanced coding, and communication techniques to secure wireless communications at the physical layer. There are two key advantages of PLS. Firstly, it enables the use of resources available at the physical layer such as multiple measurements, channel training mechanisms, power, and rate control, which cannot be utilized by the upper layers of the protocol stack. Secondly, it is based on an information-theoretic foundation for secrecy and privacy that does not make assumptions on the computational capabilities of adversaries, unlike cryptographic primitives. By considering the security and privacy requirements of recent digital systems and the potential benefits from information-theoretic security and privacy methods, it can be seen that information-theoretic methods can complement or even replace conventional cryptographic protocols for wireless networks, databases, and user authentication and identification. Since information-theoretic methods do not generally require pre-shared secret keys, they might considerably simplify the key management in complicated networks. Thus, these methods might be able to fulfill the stringent hardware area constrains of digital devices and delay constraints in 5G/6G applications, or to avoid unnecessary computations, increasing the battery life of low power devices. Information-theoretic methods offer “built-in” secrecy and privacy, generally independent of the network infrastructure, providing better scalability with respect to an increase in the network or data size.

**Figure 1:**
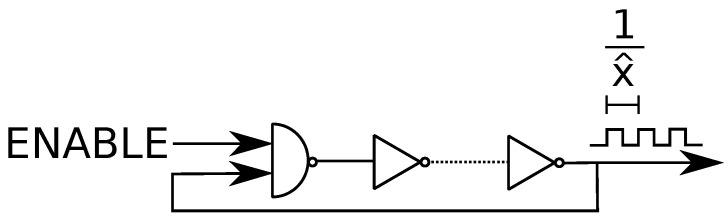
Ring oscillator (RO) logic circuit.

**Figure 2:**
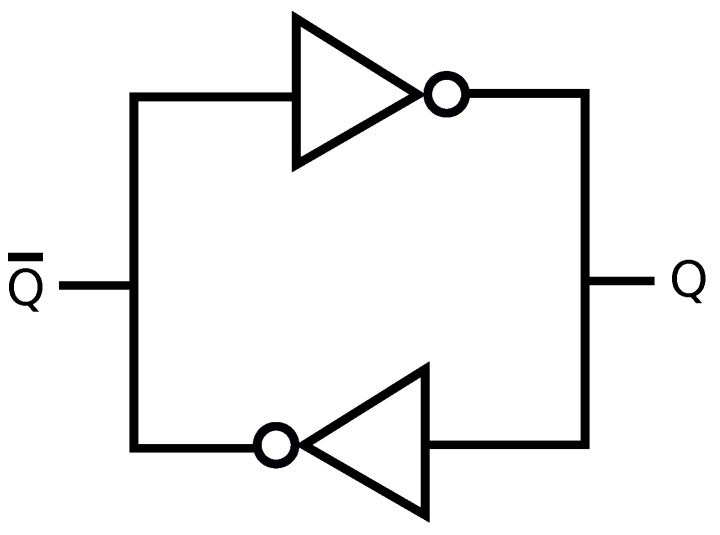
Static random access memory (SRAM) logic circuit.

A promising local solution to information-theoretic security and privacy problems is a physical unclonable function (PUF) [5]. PUFs generate “fingerprints” for physical devices by using their intrinsic and unclonable properties. For instance, consider ring oscillators (ROs) with a logic circuit of multiple inverters serially connected with a feedback of the output of the last inverter into the input of the first inverter, as depicted in Figure 1. RO outputs are oscillation frequencies $1/\hat{x}$, where $\hat{x}$ is the oscillation period, that are unique and uncontrollable since the difference between different RO outputs is caused by submicron random manufacturing variations that cannot be controlled. One can use RO outputs as a source of randomness, called a PUF circuit, to extract secret keys that are unique to the digital device that embodies these ROs. The complete method that puts out a unique secret key by using RO outputs is called an RO PUF. Similarly, binary static random access memory (SRAM) outputs are utilized as a source of randomness to implement SRAM PUFs in almost all digital devices because most digital devices have embedded SRAMs used for data storage. The logic circuit of an SRAM is depicted in Figure 2 and the logically stable states of an SRAM cell are $\left(\bar{Q},Q\right)=\left(1,0\right)$ and (0,1). During the power-up, the state is undefined if the manufacturer did not fix it. The undefined power-up state of an SRAM cell converges to one of the stable states due to random and uncontrollable mismatch of the inverter parameters, fixed when the SRAM cell is manufactured [6]. There is also random noise in the cell that affects the cell at every power-up. Since the physical mismatch of the cross-coupled inverters is due to manufacturing variations, an SRAM cell output during power-up is a PUF output that is a response with one challenge, where the challenge is the address of the SRAM cell [6].

PUFs resemble biometric features of human beings. In this review, we will list state-of-the-art methods that bridge the gap between the practical secrecy systems that use PUFs and the information-theoretic security limits by

- Modeling real PUF outputs to solve security problems with valid assumptions;
- Analyzing methods that make information-theoretic analysis tractable, for example, by transforming PUF symbols so that the transform-domain outputs are almost independent and identically distributed (i.i.d.), and that result in smaller hardware area than benchmark designs in the literature;
- Stating the information-theoretic limits for realistic PUF output models and providing optimal and practical (i.e., low-complexity and finite-length) code constructions that achieve these limits;
- Illustrating best-in-class nested codes for realistic PUF output models.

In short, we start with real PUF outputs to obtain mathematically-tractable models of their behavior and then list optimal code constructions for these models. Since we discuss methods developed from the fundamentals of signal processing and information theory, any further improvements in this topic are likely to follow the listed steps in this review.

### Organization and Main Insights

In Section 2, we provide a definition of a PUF, list its existing and potential applications, and analyze the most promising PUF types. The PUF output models and design challenges faced when manufacturing reliable, low-complexity, and secure PUFs are listed in Section 3. The main security challenge in designing PUFs, i.e., output correlations, is tackled in Section 4 mainly by using a transform coding method, which can provably protect PUFs against various machine learning attacks. The reliability and secrecy performance (e.g., the number of authenticated users) metrics used for PUF designs are defined and jointly optimized in Section 5. PUF security and complexity performance evaluations for the defined transform coding method are given in Section 6. Performance results for error-correction codes used in combination with previous code constructions that are used for key extraction with PUFs, are shown in Section 7 in order to illustrate that previous key extraction methods are strictly suboptimal. We next define the information theoretic metrics and the ultimate key-leakage-storage rate regions for the key agreement with PUFs problem, as well as comparing available code constructions for the key agreement problem in Section 8. Optimal code constructions for the key extraction with PUFs are implemented in Section 9 by using nested polar codes, which are used in 5G networks in the control channel, to illustrate significant gains from using optimal code constructions. In Section 10, we provide a list of open PUF problems that might be interesting for information theorists, coding theorists, and signal processing researchers in addition to the PUF community.

## 2. PUF Basics

We give a brief review of the literature on PUFs and discuss the problems with previous PUF designs that can be tackled by using signal processing and coding-theoretic methods.

A PUF is defined as an unclonable function embodied in a device. In the literature, there are alternative expansions of the term PUF such as “physically unclonable function”, suggesting that it is a function that is only physically-unclonable. Such PUFs may provide a weaker security guarantee since they allow their functions to be digitally-cloned. For any practical application of a PUF, we need the property of unclonability both physically and digitally. We therefore consider a function as a PUF only when the function is a physical function, i.e. it is in a device, and it is not possible to clone it physically and digitally.

Physical identifiers such as PUFs are heuristically defined to be complex challenge-response mappings that depend on the random variations in a physical object. Secret sequences are derived from this complex mapping, which can be used as a secret key. One important feature of PUFs is that the secret sequence generated is not required to be stored and it can be regenerated on demand. This property makes PUFs cheaper (no requirement for a memory for secret storage) and safer (the secret sequence is regenerated only on demand) alternatives to other secret generation and storage techniques such as storing the secret in an NVM [5].

There is an immense number of PUF types, which makes it practically impossible to give a single definition of PUFs that covers all types. We provide the following definition of PUFs that includes all PUF types of interest for this review.

**Definition** **1** ([5])**.** *We define a PUF as a challenge-response mapping embodied by a device such that it is fast and easy for the device to put out the PUF response and hard for an attacker, who does not have access to the PUF circuits, to determine the PUF output to a randomly chosen input, given that a set of challenge-response (or input-output) pairs is accessible to him.*

The terms used in Definition 1, i.e., fast, easy, and hard, are relative terms that should be quantified for each PUF application separately. There are physical functions, called physical one-way functions (POWFs), in the literature that are closely related to PUFs. Such functions are obtained by applying the cryptographic method of “one-way functions”, which refers to easy to evaluate and (on average) difficult to invert functions [7], to physical systems. As the first example of POWFs, the pattern of the speckle obtained from waves that propagate through a disordered medium is a one-way function of both the physical randomness in the medium and the angle of the beam used to generate the optical waves [8].

Similar to POWFs, biometric identifiers such as the iris, retina, and fingerprints are closely related to PUFs. Most of the assumptions made for biometric identifiers are satisfied also by PUFs, so we can apply almost all of the results in the literature for biometric identifiers to PUFs. However, it is common practice to assume that PUFs can resist invasive (physical) attacks, which are considered to be the most powerful attacks used to obtain information about a secret in a system, unlike biometric identifiers that are constantly available for attacks. The reason for this assumption is that invasive attacks permanently destroy the fragile PUF outputs [5]. This assumption will be the basis for the PUF system models used throughout this review. We; therefore, assume that the attacker does not observe a sequence that is correlated with the PUF outputs, unlike biometric identifiers, since physical attacks applied to obtain such a sequence permanently change the PUF outputs.

### 2.1. Applications of PUFs

A PUF can be seen as a source of random sequences hidden from an attacker who does not have access to the PUF outputs. Therefore, any application that takes a secret sequence as input can theoretically use PUFs. We list some scenarios where PUFs fit well practically:

- Security of information in wireless networks with an eavesdropper, i.e., a passive attacker, is a PLS problem. Consider Wyner’s wiretap channel model introduced in [9]. This model is the most common PLS model, which is a channel coding problem unlike the secret key agreement problem we consider below that is a source coding problem. A randomized encoder helps the transmitter in keeping the message secret by confusing the eavesdropper. Therefore, at the WTC transmitter, PUFs can be used as the local randomness source when a message should be sent securely through the wiretap channel.
- Consider a 5G/6G mobile device that uses a set of SRAM outputs, which are available in mobile devices, as PUF circuits to extract secret keys so that the messages to be sent are encrypted with these secret keys before sending the data over the wireless channel. Thus, the receiver (e.g., a base station) that previously obtained the secret keys (sent by mobile devices, e.g., via public key cryptography) can decrypt the data, while an eavesdropper who only overhears the data broadcast over the wireless channel cannot easily learn the message sent.
- The controller area network (CAN) bus standard used in modern vehicles is illustrated in [10] to be susceptible to denial-of-service attacks, which shows that safety-critical inputs of the internal vehicle network such as brakes and throttle can be controlled by an attacker. One countermeasure is to encrypt the transmitted CAN frames by using block ciphers with secret keys generated from PUF outputs used as inputs.
- IoT devices such as wearable or e-health devices may carry sensitive data and use a PUF to store secret keys in such a way that only a device to which the secret keys are accessible can command the IoT devices. One common example of such applications is when PUFs are used to authenticate wireless body sensor network devices [11].
- Cloud storage requires security to protect users’ sensitive data. However, securing the cloud is expensive and the users do not necessarily trust the cloud service providers. A PUF in a universal serial bus (USB) token, i.e., Saturnus^®^, has been trademarked to encrypt user data before uploading the data to the cloud, decrypted locally by reconstructing the same secret from the same PUF.
- System developers want to mutually authenticate a field programmable gate array (FPGA) chip and the intellectual property (IP) components in the chip, and IP developers want to protect the IP. In [12], a protocol is described to achieve these goals with a small hardware area that uses one symmetric cipher and one PUF.

Other applications of PUFs include providing non-repudiation (i.e., undeniable transmission or reception of data), proof of execution on a specific processor, and remote integrated circuit (IC) enabling. Every application of PUFs has different assumptions about the PUF properties, computational complexity, and the specific system models. Therefore, there are different constraints and system parameters for each application. We focus mainly on the application where a secret key is generated from a PUF for user, or device, authentication with privacy and secrecy guarantees, and low complexity.

### 2.2. Main PUF Types

We review four PUF types, i.e., silicon, arbiter, RO, and SRAM PUFs. We consider mainly the last two PUF types for algorithm and code designs due to their common use in practice and because signal processing techniques can tackle the problems arising in designing these PUFs. For a review of other PUF types that are mostly considered in the hardware design and computer science literatures, and various classifications of PUFs, see, for example, [4,13,14]. The four PUF types considered below can be shown to satisfy the assumption that invasive attacks permanently change PUF outputs, since digital circuit outputs used as the source of randomness in these PUF types change permanently under invasive attacks due to their dependence on nano-scale alterations in the hardware.

### 2.3. Silicon and Arbiter PUFs

Common complementary metal-oxide-semiconductor (CMOS) manufacturing processes are used to build silicon PUFs, where the response of the PUF depends on the circuit delays, which vary across integrated circuits (ICs) [5]. Due to high sensitivity of the circuit delays to environmental changes (e.g., ambient temperature and power supply voltage), arbiter PUFs are proposed in [15], for which an arbiter (i.e., a simple transparent data latch) is added to the silicon PUFs so that the delay comparison result is a single bit. The difference of the path delays is mapped to, for example, the bit 0 if the first path is faster, and the bit 1 otherwise. The difference between the delays can be small, causing meta-stable outputs. Since the output of the mapper is generally pre-assigned to the bit 0, the signals that are incoming are required to satisfy a setup time ($t_{\mathrm{setup}}$), required by the latch to change the output to the bit 1, resulting in a bias in the arbiter PUF outputs. Symmetrically implementable latches (e.g., set-reset latches) should be used to overcome this problem, which is difficult because FPGA routing does not allow the user to enforce symmetry in the hardware implementation. We discuss below that PUFs without symmetry requirements, for example, RO PUFs, provide better results.

### 2.4. RO PUFs

The RO logic circuit is depicted in Figure 1, where an odd number of inverters are connected serially with feedback. The first logic gate in Figure 1 is a NAND gate, giving the same logic output as an inverter gate when the ENABLE signal is 1 (ON), to enable/disable the RO circuit. The manufacturing-dependent and uncontrollable component in an RO is the total propagation delay of an input signal to flow through the RO, determining the oscillation frequency $\frac{1}{\hat{x}}$ of an RO that is used as the source of randomness. A self-sustained oscillation is possible when the ring that oscillates at the oscillation frequency $\frac{1}{\hat{x}}$ of the RO provides a phase shift of 2π with a voltage gain of 1.

Consider an RO with m≥3 inverters. Each inverter should provide a phase shift of $\frac{\pi }{m}$ with an additional phase shift of π due to the feedback. Therefore, the signal should flow through the RO twice to provide the necessary phase shift [16]. Suppose a propagation delay of $\tau_{d}$ for each inverter, so the oscillation frequency of an RO is $\frac{1}{\hat{x}}=\frac{1}{2m\tau_{d}}$. We remark that since RO outputs are generally measured by using 32-bit counters, it is realistic to assume that a measured RO output $\frac{1}{\hat{x}}$ is a realization of a continuous distribution that can be modeled by using the histogram of a family of RO outputs with the same circuit design, as assumed below.

**Figure 3:**
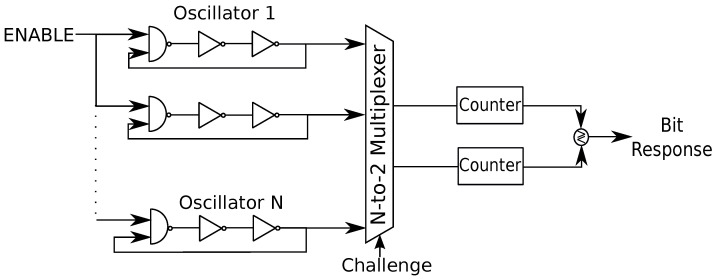
The first and most common RO physical unclonable function (PUF) design [17].

The propagation delay $\tau_{d}$ is affected by nonlinearities in the digital circuit. Furthermore, there are deterministic and additional random noise sources [16]. Such effects should be eliminated to have a reliable RO output. Rather than improving the standard RO designs, which would impose the condition that manufacturers should change their RO designs, the first proposal to fix the reliability problem was to make hard bit decisions by comparing RO pairs [17], as illustrated in Figure 3.

In Figure 3, the multiplexers are challenged by a bit sequence of length at most $\left\lceil \log_{2}N\right\rceil$ so that an RO pair out of *N* ROs is selected. The counters count the number of times a rising edge is observed for each RO during a fixed time. A logic bit decision is made by comparing the counter values, which can be bijectively mapped to the oscillation frequencies. For instance, when the upper RO has a greater counter value, then the bit 0 is generated; otherwise, the bit 1. Given that ROs are identically laid out in the hardware, the differences in the oscillation frequencies are determined mainly by uncontrollable manufacturing variations. Furthermore, it is not necessary to have a symmetric layout when hard-macro hardware designs are used for different ROs, unlike arbiter PUFs.

The key extraction method illustrated in Figure 3 gives an output of N2 bits, which are correlated due to overlapping RO comparisons. This causes a security threat and makes the RO PUF vulnerable to various attacks, including machine learning attacks. Thus, non-overlapping pairs of ROs are used in [17] to extract each bit. However, there are systematic variations in the neighboring ROs due to the surrounding logic, which also should be eliminated to extract sequences with full entropy. Furthermore, ambient temperature and supply voltage variations are the most important effects that reduce the reliability of RO PUF outputs. A scheme called *1-out-of-k masking* is proposed as a countermeasure to these effects, which compares the RO pairs that have the maximum difference between their oscillation frequencies for a wide range of temperatures and voltages to extract bits [17]. The bits extracted by such a comparison are more reliable than the bits extracted by using previous methods. The main disadvantages of this scheme are that it is inefficient due to unused RO pairs, and only a single bit is extracted from the (semi-) continuous RO outputs. We review transform-coding based RO PUF methods below that significantly improve on these methods without changing the standard RO hardware designs.

### 2.5. SRAM PUFs

There are multiple memory-based PUFs such as SRAM, Flip-flop, DRAM, and Butterfly PUFs. Their common feature is to possess a small number of challenge-response pairs with respect to their sizes. As the most promising memory-based PUF type that is already used in the industry, we consider SRAM PUFs that use the uncontrollable settling state of bi-stable circuits [18]. In the standard SRAM design, there are four transistors used to form the logic of two cross-coupled inverters, as depicted in Figure 2, and two other transistors to access the inverters. The power-up state, i.e., $\left(\bar{Q},Q\right)=\left(1,0\right)$ or (0,1), of an SRAM cell provides one secret bit. Concatenating many such bits allows to generate a secret key from SRAM PUFs on demand. We provide an open problem about SRAM PUFs in Section 10.

## 3. Correlated, Biased, and Noisy PUF Outputs

PUF circuit outputs are biased (nonuniform), correlated (dependent), and noisy (erroneous). We review a transform-coding algorithm that extracts an almost i.i.d. uniform bit sequence from each PUF, so a helper-data generation algorithm can correct the bit errors in the sequence generated from noisy PUF outputs. Using this transform-coding algorithm, we also obtain memoryless PUF measurement-channel models, so standard information-theoretic tools, which cannot be easily applied to correlated sequences, can be used.

**Remark** **1.** *The bias in the PUF circuit outputs is considered in the PUF literature to be a big threat against the security of the key generated from PUFs since the bias allows to apply, for example, machine learning attacks. However, it is illustrated in [19] (Figure 6) that the output bias does not change the information-theoretic rate regions significantly, illustrating that there exist code constructions that do not require PUF outputs to be uniformly distributed.*

We consider two scenarios, where a secret key is either generated from PUF outputs (i.e., generated secret [GS] model) or they are bound to PUF outputs (chosen secret [CS] model). An example of GS methods is code-offset fuzzy extractors (COFE) [20], and an example of the CS methods is the fuzzy-commitment scheme (FCS) [21]. We first analyze a method that significantly improves privacy, reliability, hardware cost and secrecy performance, by transforming the PUF outputs into a frequency domain, which are later used in the FCS. We remark that the information-theoretic analysis of the CS model follows directly from the analysis of the GS model [22], so one can use either model for comparisons.

PUF output correlations might cause information leakage about the PUF outputs (i.e., privacy leakage) and about the secret key (i.e., secrecy leakage) [22,23]. Furthermore, channel codes are required to satisfy the constraint on the reliability due to output noise. The transform coding method proposed in [24] adjusts the PUF output noise to satisfy the reliability constraint in addition to reducing the PUF output correlations.

### 3.1. PUF Output Model

Consider a (semi-)continuous output physical function such as an RO output as a source with real valued outputs $\hat{x}$. Since in a two-dimensional (2D) array the maximum distance between RO hardware logic circuits is less than in a one-dimensional array, decreasing the variations in the RO outputs caused by surrounding hardware logic circuits [25], we consider a 2D RO array of size l=r×c that can be represented as a vector random variable ${\hat{X}}^{l}$. Each device embodies a single 2D RO array that has the same circuit design and we have ${\hat{X}}^{l}∼f_{{\hat{X}}^{l}}$, where $f_{{\hat{X}}^{l}}$ is a probability density function. Mutually independent and additive Gaussian noise denoted as ${\hat{Z}}^{l}$ disturbs the RO outputs, i.e., we have noisy RO outputs ${\hat{Y}}^{l}\;=\;{\hat{X}}^{l}\;+\;{\hat{Z}}^{l}$. Since ${\hat{X}}^{l}$ and ${\hat{Y}}^{l}$ are dependent, using these outputs a secret key can be agreed [26,27].

**Remark** **2.** *PUF outputs are noisy, as discussed above in this section. However, the first PUF outputs are used by, for example, a manufacturer to generate or embed a secret key, which is called the enrollment procedure. Since a manufacturer can measure multiple noisy outputs of the same RO to estimate the noiseless RO output, we can consider that the PUF outputs measured during enrollment are noiseless. However, during the reconstruction step, for example, an IoT device observes a noisy RO output, which can be the case because the IoT device cannot measure the RO outputs multiple times due to delay and complexity constraints. Therefore, we consider a key-agreement model where the first measurement sequence (during enrollment) is noiseless and the second measurement sequence (during reconstruction) is noisy; see also Section 8. Extensions to key agreement models with two noisy sequences, where the noise components can be correlated, are discussed in [23,28,29].*

We extract i.i.d. symbols from ${\hat{X}}^{l}$ and ${\hat{Y}}^{l}$ such that information theoretic tools used in [30] for the FCS can be applied. An algorithm is proposed in [24] to obtain almost i.i.d. uniformly-distributed and binary vectors $X^{n}$ and $Y^{n}$ from ${\hat{X}}^{l}$ and ${\hat{Y}}^{l}$, respectively. For such $X^{n}$ and $Y^{n}$, we can define a binary error vector as $E^{n}\;=\;X^{n}\;⊕\;Y^{n}$, where ⊕ is the modulo-2 sum. We then obtain the random sequence $E^{n}∼{\mathrm{Bernoulli}}^{n}\left(p\right)$, so the channel $P_{Y|X}∼\mathrm{BSC}\left(p\right)$ is a binary symmetric channel (BSC) with crossover probability *p*. We discuss a transform-coding method below, which further provides reliability guarantees for each bit generated.

**Figure 4:**
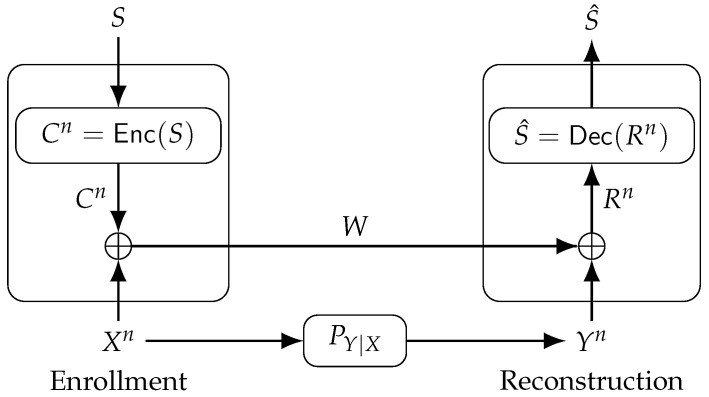
The fuzzy commitment scheme (FCS).

The FCS can reconstruct a secret key from dependent random variables with zero secrecy leakage [21]. For the FCS, depicted in Figure 4, an encoder Enc(·) maps a secret key S∈S, which is uniformly distributed in the set {1,2,…,|S|}, into a codeword $C^{n}$ with binary symbols that are later added to the PUF-output sequence $X^{n}$ in modulo-2 during enrollment. The output is called helper data W, sent to a database via a noiseless, public and authenticated communication link. The sum of *W* and $Y^{n}$ in modulo-2 is $R^{n}=W⊕Y^{n}=C^{n}\;⊕E^{n}$, mapped to a secret key estimate $\hat{S}$ during reconstruction by the decoder Dec(·).

We next give information-theoretic rate regions for the FCS; see [31] for information-theoretic notation and basics.

**Definition** **2.** *The FCS can achieve a secret-key vs. privacy-leakage rate pair $\left(R_{s}\;\;,\;\;R_{\ell }\right)$ with zero secrecy leakage (i.e., perfect secrecy) if, given any δ>0, there is some n≥1, and an encoder and decoder pair for which we have $R_{s}=\frac{\log|S|}{n}$ and*

(1) $$\begin{array}{cccc} & \mathrm{Pr}\left[\hat{S}\neq S\right]=P_{B}\leq \delta & & \left(\mathrm{reliability}\right) \end{array}$$

(2) $$\begin{array}{cccc} & H\left(S\right)\geq n\left(R_{s}-\delta \right) & & \left(\mathrm{key}\;\mathrm{uniformity}\right) \end{array}$$

(3) $$\begin{array}{cccc} & I\left(S;W\right)\;=\;0 & & \left(\mathrm{perfect}\;\mathrm{secrecy}\right) \end{array}$$

(4) $$\begin{array}{cccc} & I\left(X^{n};W\right)\leq n\left(R_{\ell }+\delta \right)\;\;\; & & \left(\mathrm{privacy}\right) \end{array}$$

*where (3) suggests that S and W are independent and (4) suggests that the rate of dependency between $X^{n}$ and W is bounded. The achievable secret-key vs. privacy-leakage rate, or key-leakage, region $R_{\mathrm{FCS}}$ for the FCS is the union of all achievable pairs.*

**Theorem** **1** ([30])**.** *The key-leakage region $R_{\mathrm{FCS}}$ for the FCS with perfect secrecy, uniformly-distributed X and Y, and a channel $P_{Y|X}∼\mathrm{BSC}\left(p\right)$ is*

(5) $$\begin{array}{cc} R_{\mathrm{FCS}}= & \{\left(R_{s},R_{\ell }\right):\;\;\;0\leq R_{s}\leq 1-H_{b}\left(p\right),\;R_{\ell }\geq 1-R_{s}\} \end{array}$$

*where $H_{b}\left(p\right)=-p\log p-\left(1-p\right)\log\left(1-p\right)$ is defined as the binary entropy function.*

The region R of all achievable (secret-key, privacy-leakage) rate pairs for the CS model with a negligible secrecy-leakage rate is [22]

(6) $$\begin{array}{cc} R\;=\; & \underset{P_{U|X}}{⋃}\;\left\{\left(R_{s},R_{\ell }\right)\;:\;\;0\leq R_{s}\leq I\left(Y;U\right),\;R_{\ell }\geq I\left(X;U\right)-I\left(Y;U\right)\right\} \end{array}$$

such that U−X−Y forms a Markov chain and it suffices to have |U|≤|X|+1. The auxiliary random variable *U* represents a distorted version of *X* through a channel $P_{U|X}$. The FCS is optimal only at the point $\left(R_{s}^{*},R_{\ell }^{*}\right)\;=\;\left(1\;-\;H_{b}\left(p\right),H_{b}\left(p\right)\right)$ [30], corresponding to the maximum secret-key rate.

## 4. Transformation Steps

**Figure 5:**
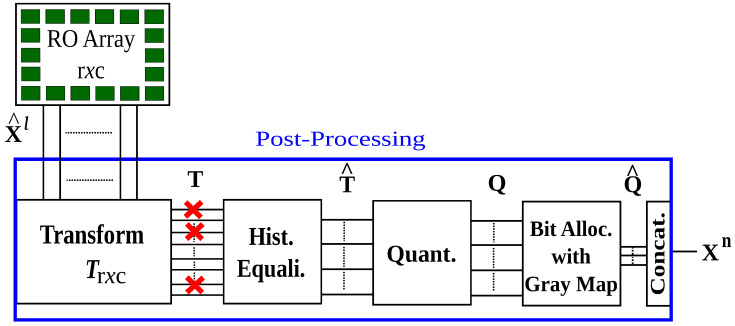
Transformation steps [24].

Transform coding methods decrease RO output correlations for ROs that are in the same 2D array by using, for example, a linear transformation. We discuss a transform-coding algorithm proposed in [32] as an extension of [24] to provide reliability guarantees to each generated bit. Joint optimization of the error-correction code and quantizer in order to maximize the reliability and secrecy are the main steps. The output of these post-processing steps is a bit sequence $X^{n}$ (or its noisy version $Y^{n}$) utilized in the FCS. It suffices to discuss only the enrollment steps, depicted in Figure 5, since the same steps are used also for reconstruction.

${\hat{X}}^{l}$ are correlated RO outputs, where the cause of correlations is, for example, the surrounding logic in the hardware. A transform $T_{r\;\times \;c}\left(\cdot \right)$ with size r×c transforms RO outputs to decrease output correlations. We model each output *T* in the transform domain, i.e., *transform coefficient*, calculated by transforming the RO outputs given in the dataset [33] by using the Bayesian information criterion (BIC) [34] and the corrected Akaike’s information criterion (AICc) [35], suggesting a Gaussian distribution as a good fit for the discrete Haar transform (DHT), discrete Walsh–Hadamard transform (DWHT), DCT, and Karhunen–Loève transform (KLT).

In Figure 5, the histogram equalization changes the probability density of the *i*-th coefficient $T_{i}$ into a standard normal distribution so that quantizers are the same for all transform coefficients, decreasing the storage. Obtained coefficients ${\hat{T}}_{i}$ are independent when the transform coefficients $T_{i}$ are jointly Gaussian and the transform $T_{r\;\times \;c}\left(\cdot \right)$ decorrelates the RO outputs perfectly. For such a case, scalar quantizers do not introduce any performance loss. Bit extraction methods and scalar quantizers are given below for the FCS with the independence assumption, which can be combined with a correlation-thresholding approach in practice.

## 5. Joint Quantizer and Error-Correction Code Design

The steps in Figure 5 are applied to obtain a uniform binary sequence $X^{n}$. We utilize a quantizer Δ(·) that assigns quantization-interval values of $k=1,2,\cdots ,2^{K_{i}}$, where $K_{i}$ represents the number of bits obtained from the *i*-th coefficient. We have

(7) $$\begin{array}{c} \Delta \left({\hat{t}}_{i}\right)=k\;\mathrm{if}\;b_{k-1}\;<\;{\hat{t}}_{i}\;\leq \;b_{k} \end{array}$$

where we have $b_{k}=\Phi^{-1}\left(\frac{k}{2^{K_{i}}}\right)$, and $\Phi^{-1}\left(\cdot \right)$ is the standard Gaussian distribution’s quantile function. A length-$K_{i}$ bit sequence represents the output *k*. Since the noise has zero mean, we use a Gray mapping to determine the sequences assigned to each *k*, so neighboring sequences differ only in one bit.

### Quantizers with Given Maximum Number of Errors

We discuss a conservative approach that suppose either bits assigned to a quantized transform coefficient all flip or they are all correct. Let the correctness probability $P_{c}$ of a coefficient be the probability that all bits assigned to a transform coefficient are correct, used to choose the number of bits extracted from a coefficient in such a way that one can design a channel encoder with a bounded minimum distance decoder (BMDD) to satisfy the reliability constraint $P_{B}\leq 10^{-9}$, a common value for the block-error probability of PUFs that use CMOS circuits [17].

Let Q(·) be the Q-function, $f_{\hat{T}}$ the probability density of the standard Gaussian distribution, and $\sigma_{\hat{n}}^{2}$ the noise variance. The correctness probability can be calculated as

(8) $$\begin{array}{c} P_{c}\left(K\right)\;=\;\sum_{k=0}^{2^{K}-1}\;\int_{b_{k}}^{b_{k+1}}\left[\;Q\left(\frac{b_{k}\;-\;\hat{t}}{\sigma_{\hat{n}}}\right)\;-\;Q\left(\frac{b_{k+1}\;-\;\hat{t}}{\sigma_{\hat{n}}}\right)\;\right]f_{\hat{T}}\left(\hat{t}\right)d\hat{t} \end{array}$$

where *K* is the length of the bit sequence assigned to a quantizer with quantization boundaries $b_{k}$ from (7) for an equalized Gaussian transform coefficient $\hat{T}$. In (8), we calculate the probability that the additive noise will not change the quantization interval assigned to the transform coefficient, i.e., all bits associated with the transform coefficient stay the same after adding noise.

Assume that all errors in up to $C_{\max}$ coefficients can be corrected by a channel decoder, that the correctness probability $P_{c,i}\left(K\right)$ of the *i*-th coefficient ${\hat{T}}_{i}$ is greater than or equal to $\bar{P}\left(C_{\max}\right)$, and that errors occur independently. We first find the minimum correctness probability that satisfies $P_{B}\;\leq \;10^{-9}$, denoted as $\bar{P}\left(C_{\max}\right)$, by solving

(9) ∑c=Cmax+1llc(1−P¯c(Cmax))cP¯c(Cmax)l−c≤10−9

which allows to find the maximum bit-sequence length $K_{i}$ for the *i*-th transform coefficient such that $P_{c,i}\left(K\right)\geq {\bar{P}}_{c}\left(C_{\max}\right)$. The first transform coefficient, i.e., DC coefficient, ${\hat{T}}_{1}$ can in general be estimated by an attacker, which is the first reason why it is not used for key extraction. As the second reason, temperature and voltage changes affect RO outputs highly linearly, which affects the DC coefficient the most [36]. Thus, we fix $K_{1}\;=\;0$, so the total number of extracted bits can be calculated as

(10) $$\begin{array}{c} n\left(C_{\max}\right)\;=\;\sum_{i=2}^{l}K_{i}. \end{array}$$

We first sort $K_{i}$ values in descending order such that $K_{i}^{\prime }\;\geq \;K_{i+1}^{\prime }$ for all i=1,2,…,l−2. Thus, up to

(11) $$\begin{array}{c} e\left(C_{\max}\right)=\sum_{i=1}^{C_{\max}}K_{i}^{\prime } \end{array}$$

bit errors must be corrected for the worst case scenario. Using a BMDD, a block code with minimum distance $d_{\min}\;\geq \;2e\left(C_{\max}\right)\;+\;1$ can satisfy this requirement [37].

The advanced encryption standard (AES) requires a seed of, e.g., a secret key with length 128 bits. If the FCS is applied to PUFs to extract such a secret key for the AES, the block code designed should have a code length $\leq n(C_{\max})$ bits, code dimension ≥128 bits, and minimum distance $d_{\min}\geq 2e\left(C_{\max}\right)+1$, given a $C_{\max}$. Such an optimization problem is generally hard to solve but, using an exhaustive search over different $C_{\max}$ values and over different algebraic codes, one can show the existence of a channel code that satisfies all constraints. Considering codes with low-complexity implementations is preferred for, e.g., IoT applications. We remark that the correctness probability might be significantly greater than ${\bar{P}}_{c}\left(C_{\max}\right)$, that the probability that less than $K_{i}$ bits are actually in error when the *i*-th coefficient is erroneous is high, and that the bit errors do not necessarily happen in the coefficients from which the maximum-length bit sequences are obtained. Therefore, we next illustrate that even though $e(C_{\max})$ errors cannot be corrected, the constraint $P_{B}\;\leq \;10^{-9}$ is satisfied.

## 6. PUF Performance Evaluations

Represent RO outputs ${\hat{X}}^{l}$ as a vector random variable with the autocovariance matrix $C_{\hat{X}\hat{X}}$ and consider 8 × 8 and 16 × 16 RO arrays, whose autocovariance matrix is estimated by using the RO outputs in [33]. Using the dataset, we next compare the performance of the DWHT, DCT, KLT, and DHT in terms of their security, decorrelation efficiency, uniqueness, and complexity.

### 6.1. Decorrelation Efficiency

Consider the autocovariance matrix $C_{\mathrm{TT}}$ of the transform coefficients so that the decorrelation efficiency $\eta_{c}$, used as a decorrelation performance metric, of a fixed transform is [38]

(12) $$\begin{array}{c} \eta_{c}=1-\frac{\sum_{a=1}^{l}\sum_{b=1}^{l}\left|C_{\mathrm{TT}}\left(a,b\right)\right|𝟙\left\{a\;\neq \;b\right\}}{\sum_{a=1}^{l}\sum_{b=1}^{l}\left|C_{\hat{X}\hat{X}}\left(a,b\right)\right|𝟙\left\{a\;\neq \;b\right\}} \end{array}$$

where 𝟙{·} is the indicator function. The KLT has a decorrelation efficiency of 1, i.e., optimal [38]. Average $\eta_{c}$ values of remaining transforms are given in Table 1 and they have good (i.e., high) and similar decorrelation efficiency performance. The DHT and DCT have the highest efficiency for 8×8 RO arrays; while, for 16×16 RO arrays, the DWHT is the best transform. Table 1 suggests that an array size increase improves $\eta_{c}$.

### 6.2. Complexity of Transforms

Computational complexity of r×c=8×8 and 16×16 RO arrays are considered, which are powers of 2 so that there are fast algorithms to implement the DWHT, DCT, and DHT. The KLT has a computational complexity of $O(n^{3})$ for r=c=n; while, the DWHT and DCT have $O(n^{2}\log_{2}n)$, and the DHT has $O(n^{2})$ [39]. Efficient implementations of the DWHT that do not require multiplications exist [32], which can be applied also to the transforms proposed in [40]. The DWHT is therefore a good candidate for implementing RO PUFs for IoT applications. For instance, a hardware implementation of 2D DWHT in an evaluation board of Xilinx ZC706 with a Zynq-7000 XC7Z045 system-on-chip is illustrated in [32] to require approximately 11% smaller hardware area and 64% less processing time than the benchmark RO PUF hardware implementation in [41].

### 6.3. Security and Uniqueness

The extracted bit sequence is required to be uniformly distributed to use the rate region $R_{\mathrm{FCS}}$ in (5). The randomness measure called uniqueness is the average fractional Hamming distance between bit sequences generated from different RO PUFs. All transforms have similar uniqueness results with a mean Hamming distance of 0.500 and Hamming distance variance is $7\times 10^{-4}$. These results are close to optimal uniqueness results, expected because of equipartitioned quantization intervals and high decorrelation efficiencies, that are better than previous uniqueness results with mean values of 0.462 [17] and 0.473 [33].

The national institute of standards and technology (NIST) has randomness tests to check if an extracted binary sequence can be differentiated from a uniformly-random binary sequence [42]. The bit sequences with the DWHT pass most of the applicable tests, considered to be an acceptable result [42]. The KLT performs the best because of its optimal decorrelation performance.

## 7. Error-Correction Codes for PUFs with Transform Coding

Suppose that bit sequences extracted by using the transform-coding method are i.i.d. and uniformly distributed, so perfect secrecy is satisfied. We assume that signal processing steps mentioned above perform well, so we can conduct standard information- and coding-theoretic analysis. We provide a list of codes designed for the transform-coding algorithm by using the reliability metric considered above.

**Figure 6:**
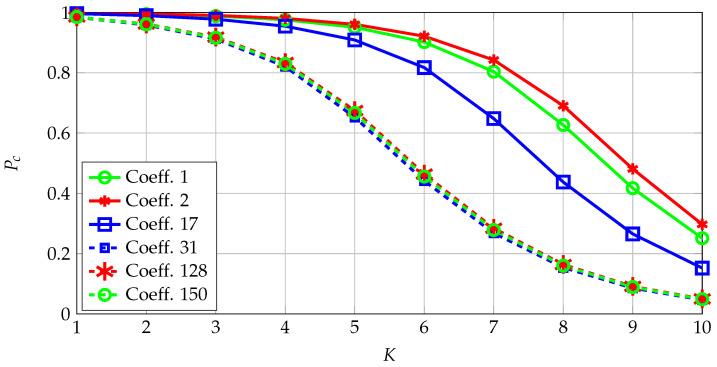
Transform coefficients’ correctness probabilities.

Select a channel code for the quantizer designed above for a fixed maximum number of errors for a secret key of size 128 bits. The correctness probabilities for the coefficients with the smallest and highest probabilities are depicted in Figure 6. Transform coefficients that represent the low-frequency coefficients are the most reliable, which are at the upper-left corner of the 2D transform-coefficient array with indices such as 1,17,2,18,3,19. These coefficients thus have the highest signal-to-noise ratios (SNRs). Conversely, the least reliable coefficients are observed to be coefficients that represent intermediate frequencies, indicating that one can define a metric called SNR-packing efficiency, defined similarly as the energy-packing efficiency, and show that it follows a more complicated scan order than the classic zig-zag scan order used for the energy-packing efficiency.

Fix $C_{\max}$, defined above, and calculate ${\bar{P}}_{c}\left(C_{\max}\right)$ via (9), $n(C_{\max})$ via (10), and $e(C_{\max})$ via (11). If $C_{\max}\;\leq \;10$, ${\bar{P}}_{c}\left(C_{\max}\right)$ is large and $P_{c,i}\left(K\;=\;1\right)\;\leq \;{\bar{P}}_{c}\left(C_{\max}\right)$ for all i=2,…,l. In addition, if $11\;\leq \;C_{\max}\;\leq \;15$, then $n(C_{\max})\leq 128$ bits. Furthermore, if $C_{\max}$ increases, ${\bar{P}}_{c}\left(C_{\max}\right)$ decreases, so the maximum of the number $K_{\max}\left(C_{\max}\right)\;=\;K_{1}^{\prime }\left(C_{\max}\right)$ of bits extracted among all used coefficients increases, increasing the hardware complexity. Thus, consider only the cases where $C_{\max}\;\leq \;20$. Table 2 shows ${\bar{P}}_{c}\left(C_{\max}\right)$, $n(C_{\max})$, and $e(C_{\max})$ for a range of $C_{\max}$ values used for channel-code selection.

Consider Reed–Solomon (RS) and binary (extended) Bose–Chaudhuri–Hocquenghem (BCH) codes, whose minimum-distance $d_{\min}$ is high. There is no BCH or RS code with parameters satisfying any of the $\left(n\left(C_{\max}\right),\;e\left(C_{\max}\right)\right)$ pairs in Table 2 such that its dimension is ≥128 bits. However, the analysis leading to Table 2 is conservative. Thus, we next find a BCH code whose parameters are as close as possible to an $\left(n\left(C_{\max}\right),\;e\left(C_{\max}\right)\right)$ pair in Table 2. Consider the binary BCH code that can correct all error patterns with up to $e_{\mathrm{BCH}}=18$ errors with the block length of 255 and code dimension of 131 bits.

First, extract exactly one bit from each transform coefficient, i.e., $K_{i}\;=\;1$ for all i=2,3,…,l, so n=l−1=255 bits are extracted, resulting in mutually-independent bit errors $E_{i}$. Thus, all error patterns with up to e=20 bit errors should be corrected by the chosen code rather than e(20)=25 bit errors. However, this value is still greater than $e_{\mathrm{BCH}}=18$.

**Figure 7:**
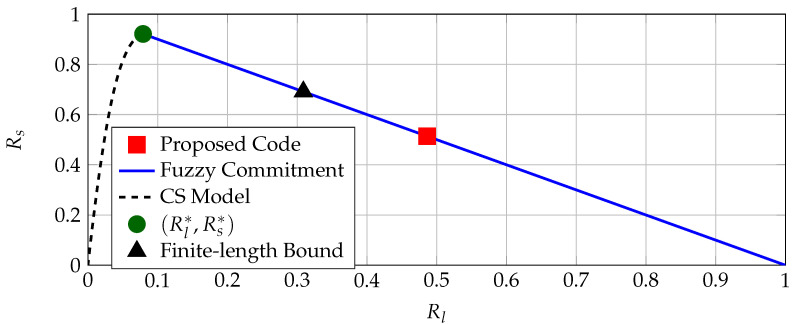
The operation point of the considered Bose–Chaudhuri–Hocquenghem (BCH) code C(255,131,37), the maximum secret-key rate point $\left(R_{\ell }^{*},\;R_{s}^{*}\right)$, regions of achievable rate pairs according to (5) and (6), and a finite-length bound for BSC(0.0097), n=255 bits, and $P_{B}=10^{-9}$.

The block error probability $P_{B}$ for the BCH code C(255,131,37) with a BMDD is equal to the probability of encountering more than 18 errors, i.e., we have

(13) $$\begin{array}{c} P_{B}=\sum_{j=19}^{255}[\sum_{A\in F_{j}}\prod_{i\in A}\left(1-P_{c,i}\right)\;\cdot \prod_{i\in A^{c}}P_{c,i}] \end{array}$$

where $P_{c,i}$ is the correctness probability of the *i*-th coefficient ${\hat{T}}_{i}$ as in (8) for i=2,3,…,256, $A^{c}$ denotes the complement of the set *A*, and $F_{j}$ is the set of all size-*j* subsets of the set {2,3,…,256}. $P_{c,i}$ values are different and they represent probabilities of independent events because we assume that the transform coefficients are independent. We apply the discrete Fourier transform characteristic function method [43] to evaluate the block-error probability with the result $P_{B}\;\;\approx \;\;1.26\;\;\times \;\;10^{-11}\;\;<\;\;10^{-9}$. The block-error probability (i.e., reliability) constraint is therefore satisfied by the BCH code C(255,131,37), although the conservative analysis suggested otherwise. This code achieves a (secret-key, privacy-leakage) rate pair of $\left(R_{s},R_{\ell }\right)=\left(\frac{131}{255},1\;-\;\frac{131}{255}\right)\approx \left(0.514,\;0.486\right)$ bits/source-bit, which is significantly better than previous results. We next consider the region of all achievable rate pairs for the CS model and the FCS for a BSC $P_{Y|X}$ with crossover probability $p_{b}\;=\;1-\frac{1}{l-1}\sum_{i=2}^{l}P_{c,i}\left(K_{i}\;\;=\;\;1\right)\;\approx \;0.0097$, i.e., probability of being in error averaged over all used coefficients with the above defined quantizer. The (secret-key, privacy-leakage) rate pair of the BCH code, regions of all rate pairs achievable by the FCS and CS model, the maximum secret-key rate point, and a finite-length bound [44] for the block length of n=255 bits and $P_{B}\;=\;10^{-9}$ are depicted in Figure 7 for comparisons.

Denote the maximum secret-key rate as $R_{s}^{*}\;\;\approx \;\;0.922$ bits/source-bit and the corresponding minimum privacy-leakage rate as $R_{\ell }^{*}\;\;\approx \;\;0.079$ bits/source-bit. The gap between $\left(R_{\ell }^{*},\;R_{s}^{*}\right)$ at which the FCS is optimal and the rate tuple achieved by the BCH code can be explained by the short block length and small block-error probability. However, the finite-length bound given in [44] (Theorem 52) suggests that the FCS can achieve the rate tuple $\left(R_{s},\;R_{\ell }\right)\;=\;\left(0.691,\;0.309\right)$ bits/source-bit, shown in Figure 7. Better channel code designs and decoders (possibly with higher hardware implementation complexity) can improve the performance, but they might not be feasible for IoT applications. Figure 7 shows that there are other code constructions (that are not standard error-correcting codes) that can achieve smaller privacy-leakage and storage rates for a fixed secret-key rate, illustrated below.

## 8. Code Constructions for PUFs

Consider the two-terminal key agreement problem, where the identifier outputs during enrollment are noiseless. We mention two optimal linear code constructions from [45] that are based on distributed lossy source coding (or Wyner–Ziv [WZ] coding) [46]. The random linear code construction achieves the GS and CS models’ key-leakage-storage regions and the nested polar code construction jointly designs vector quantization (during enrollment) and error correction (during reconstruction) codes. Designed nested polar codes improve on existing code designs in terms of privacy-leakage and storage rates, and one code achieves a rate tuple that existing methods cannot achieve.

Several practical code constructions for key agreement with identifiers have been proposed in the literature. For instance, the COFE and the FCS both require a standard error-correction code to satisfy the constraints of, respectively, the key generation (GS model) and key embedding (CS model) problems, as discussed above. Similarly, a polar code construction is proposed for the GS model in [47]. These constructions are sub-optimal in terms of storage and privacy-leakage rates.

A Golay code is used as a vector quantizer (VQ) in [22] in combination with distributed lossless source codes (or Slepian–Wolf [SW] codes) [48] to increase the ratio of key vs. storage rates (or key vs. leakage rates). Thus, we next consider VQ by using WZ coding to decrease storage rates. The WZ-coding construction turns out to be optimal, which is not coincidental. For instance, the bounds on the storage rate of the GS model and on the WZ rate (storage rate) have the same mutual information terms optimized over the same conditional probability distribution. This similarity suggests an equivalence that is closely related to the concept of formula duality. In fact, the optimal random code construction, encoding, and decoding operations are identical for both problems. One therefore can call the GS model and WZ problem functionally equivalent. Such a strong connection suggests that there might exist constructive methods that are optimal for both problems for all channels, which is closely related to the operational duality concept.

**Figure 8:**
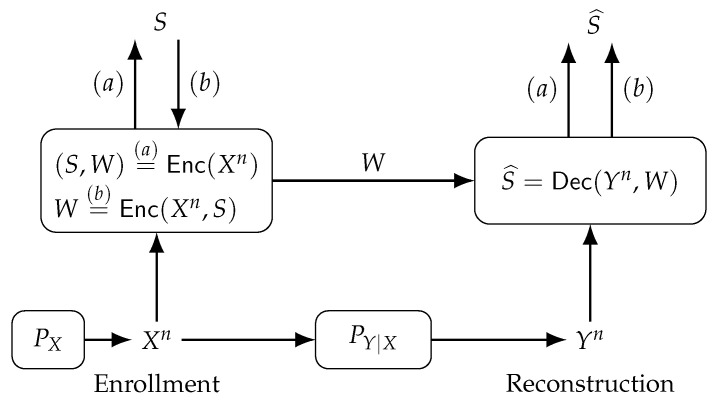
The (*a*) generated-secret (GS) and (*b*) chosen-secret (CS) models.

Consider the GS model, where a secret key is generated from a physical or biometric source, depicted in Figure 8(*a*). The encoder Enc(·) observes during enrollment the noiseless i.i.d. sequence $X^{n}∼P_{X}$ to generate public helper data *W* and a secret key *S*, i.e., $\left(S,W\right)\;=\;\mathrm{Enc}\left(X^{n}\right)$. The decoder Dec(·) observes during reconstruction the helper data *W* and a noisy measurement $Y^{n}$ of $X^{n}$ through a memoryless channel $P_{Y|X}$ to estimate the secret key, i.e., $\hat{S}\;=\;\mathrm{Dec}\left(Y^{n},\;W\right)$. Similarly, the CS model is shown in Figure 8(*b*), where a secret key *S* independent of $\left(X^{n},\;Y^{n}\right)$ is chosen and embedded into the helper data, i.e., $W=\mathrm{Enc}(X^{n},S)$. The alphabets X, Y, S, and W are finite sets, which can be achieved if, for example, the transform-coding algorithm discussed above is applied.

**Definition** **3.** *For GS and CS models, a key-leakage-storage tuple $\left(R_{s},R_{\ell },R_{w}\right)$ is* achievable *if, given any δ>0, there is an encoder, a decoder, and some n≥1 such that $R_{s}=\frac{\log|S|}{n}$ and*

(14) $$\begin{array}{cccc} & \mathrm{Pr}\left[\hat{S}\neq S\right]=P_{B}\leq \delta & & \;\;(reliability) \end{array}$$

(15) $$\begin{array}{cccc} & I\left(W;S\right)\leq n\delta & & \;\;(weak\;secrecy) \end{array}$$

(16) $$\begin{array}{cccc} & I\left(X^{n};W\right)\leq n\left(R_{\ell }+\delta \right)\;\;\; & & \;\;(privacy) \end{array}$$

(17) $$\begin{array}{cccc} & H\left(S\right)\geq n\left(R_{s}-\delta \right)\; & & \;\;(uniformity) \end{array}$$

(18) $$\begin{array}{cccc} & \log|W|\leq n(R_{w}+\delta ) & & \;\;(storage) \end{array}$$

*are satisfied. The* key-leakage-storage *regions $R_{\mathrm{gs}}$ for the GS model and $R_{\mathrm{cs}}$ for the CS model are the closures of the sets of achievable tuples for these models.*

**Theorem** **2** ([22])**.** *The key-leakage-storage regions $R_{\mathrm{gs}}$ and $R_{\mathrm{cs}}$ for the GS and CS models, respectively, are*

$$\begin{array}{c} \begin{array}{cc} R_{\mathrm{gs}}\;=\; & \underset{P_{U|X}}{⋃}\;\{\left(R_{s},R_{\ell },R_{w}\right)\;:\;\\ \; & 0\leq R_{s}\leq I\left(Y;U\right),\\ \; & R_{\ell }\geq I\left(X;U\right)-I\left(Y;U\right),\\ \; & R_{w}\geq I\left(X;U\right)-I\left(Y;U\right)\}, \end{array}\;\;\;\mathrm{and}\;\;\begin{array}{cc} R_{\mathrm{cs}}\;=\; & \underset{P_{U|X}}{⋃}\;\{\left(R_{s},R_{\ell },R_{w}\right)\;:\;\\ \; & 0\leq R_{s}\leq I\left(Y;U\right),\\ \; & R_{\ell }\geq I\left(X;U\right)-I\left(Y;U\right),\\ \; & R_{w}\geq I\left(X;U\right)\} \end{array} \end{array}$$

*where U−X−Y form a Markov chain. $R_{\mathrm{gs}}$ and $R_{\mathrm{cs}}$ are convex sets and |U|≤|X|+1 suffices for both rate regions.*

**Remark** **3.** *Improvement of the weak secrecy to strong secrecy, where (15) is replaced with I(W;S)≤δ, is possible by using multiple identifier output blocks as described in [49], e.g., by using multiple PUFs in the same device.*

Assume, as above, that $X^{n}∼{\mathrm{Bernoulli}}^{n}\left(\frac{1}{2}\right)$ and the channel $P_{Y|X}∼\mathrm{BSC}\left(p_{A}\right)$ for $p_{A}\in \left[0,0.5\right]$. Define the star-operation as $q*p_{A}=q\left(1-p_{A}\right)+\left(1-q\right)p_{A}$. The key-leakage-storage region of this GS model is

(19) $$\begin{array}{cc} R_{\mathrm{gs},\mathrm{bin}}\;=\; & \underset{q\in [0,0.5]}{⋃}\;\{\left(R_{s},R_{\ell },R_{w}\right)\;:\;\\ \; & 0\leq R_{s}\leq 1-H_{b}\left(q*p_{A}\right),\\ \; & R_{\ell }\geq H_{b}\left(q*p_{A}\right)-H_{b}\left(q\right),\\ \; & R_{w}\geq H_{b}\left(q*p_{A}\right)-H_{b}\left(q\right)\}. \end{array}$$

### Comparisons Between Code Constructions for PUFs

We consider three best code constructions proposed for the GS and CS models, which are COFE and the polar code construction in [47] for the GS model, and FCS for the CS model, in order to compare them with the WZ-coding constructions. The FCS and COFE achieve only a single point on the key-leakage rate region boundary, i.e., $R_{s}^{*}=I\left(X;Y\right)$ and $R_{\ell }^{*}=H\left(X|Y\right)$.

Adding a VQ step, one can improve these two methods. During enrollment rather than $X^{n}$, its quantized version $X_{q}^{n}$ can be used for this purpose, which can be asymptotically represented as summing the original helper data and another independent random variable $J^{n}∼{\mathrm{Bernoulli}}^{n}\left(q\right)$, i.e., $W=X^{n}⊕C^{n}⊕J^{n}$ is the (new) helper data. Modified FCS and COFE can achieve the key-leakage region when a union of all achieved rate tuples is taken over all q∈[0,0.5]. Nevertheless, the helper data of the modified FCS and COFE have length *n* bits, i.e., the storage rate is 1 bit/source-bit, which is suboptimal.

The storage rate of 1 bit/source-bit is decreased by using the polar code construction proposed in [47]. Nevertheless, this construction cannot achieve the key-leakage-storage region. In addition, in [47] there is an assumption that a “private” key that is shared between the encoder and decoder is available, which is not realistic because there is a need for hardware protection against invasive attacks to have such a private key. If such a hardware protection is feasible, there is no need to utilize an on-demand key reconstruction and storage method like a PUF. The previous methods cannot, therefore, achieve the key-leakage-storage region for a BSC, unlike the distributed lossy source coding constructions proposed in [45]. To compare such WZ-coding constructions, we use the ratio of key vs. storage rates as the metric, which determines the design procedures to control the storage and privacy leakage.

## 9. Optimal Nested Polar Code Constructions

The first channel codes with asymptotic information-theoretic optimality and low decoding complexity are polar codes [50], whose finite length performance is good when a list decoder is utilized. Nesting two codes is simple with polar codes due to their simple matrix representation; therefore, one can use them for distributed lossy source coding [51]. The *channel polarization* phenomenon, i.e., converting a channel into polarized binary channels by using a polar transform, is the core of polar codes. The polar transform takes a sequence $U^{n}$ with unfrozen and frozen bits as input and converts it into a codeword that has also length *n*. The decoder then observes a noisy codeword in addition to the fixed frozen bits of $U^{n}$ in order to estimate the bit sequence $U^{n}$. A polar code with block length *n*, and frozen bit sequence $G^{\left|F\right|}$ at indices F are denoted as $C(n,F,G^{\left|F\right|})$. We next utilize nested polar codes that are proposed for WZ coding in [51].

### 9.1. The GS Model Polar Code Construction

Consider two nested polar codes C(n,F,$\bar{V}$) and $C_{1}$(*n*, $F_{1}$, *V*) such that F = $F_{1}$ ⋃ $F_{w}$  and $\bar{V}$ = [*W*,*V*], where *W* is of length *m*_2_ and *V* is of length *m*_1_ . Suppose *m*_1_ and *m*_2_ satisfy

(20) $$\begin{array}{cc} \; & \frac{m_{1}}{n}=H_{b}\left(q\right)-\delta \end{array}$$

(21) $$\begin{array}{cc} \; & \frac{m_{1}+m_{2}}{n}=H_{b}\left(q*p_{A}\right)+\delta \end{array}$$

for a *δ* > 0 and some distortion *q* ∈ [0,0.5]. Two polar codes C(*n*,F, $\bar{V}$) and $C_{1}$(*n*,$C_{1}$,*V*) are nested since the set of indices $F_{1}$ refer to frozen channels with values *V*, which are common to both polar codes, and the code C has further frozen channels with values *W* at indices $F_{w}$.

**Figure 9:**
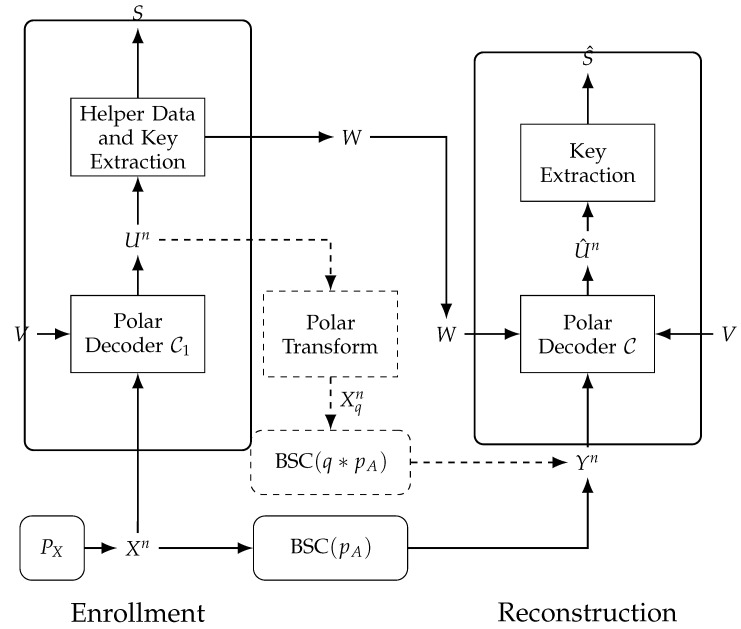
Second WZ-coding construction for the GS model.

Since the rate of $C_{1}$ is greater than the capacity of the lossy source coding problem for an average distortion *q*, it functions as a VQ with distortion *q*. Furthermore, since the rate of C is less than the channel capacity of the BSC($q*p_{A}$), it functions as an error-correcting code. We want to calculate the values *W* during enrollment, stored as the public helper data, such that $\left(V,W,Y^{n}\right)$ can be used during reconstruction to estimate the key *S* with length $n-m_{1}-m_{2}$, which is depicted in Figure 9. We assign the all-zero vector to *V*, so to not increase storage, which does not affect the average distortion E[q] between $X_{q}^{n}$ and $X^{n}$ defined below; see [51] (Lemma 10) for a proof.

During enrollment, the PUF outputs $X^{n}∼{\mathrm{Bernoulli}}^{n}\left(\frac{1}{2}\right)$ are observed by a polar decoder of $C_{1}$ and considered as noisy measurements of a sequence $X_{q}^{n}$ measured through a BSC(q), i.e., $X^{n}$ is quantized into $X_{q}^{n}$ by a polar decoder of $C_{1}$. The polar decoder puts out the sequence $U^{n}$ and the bit values *W* at its indices $F_{w}$ are publicly stored as the helper data. Furthermore, the bit values at indices j∈{1,2,…,n}\F are assigned as the secret key *S*. We remark that the polar transform of $U^{n}$ is the sequence $X_{q}^{n}$ that is the quantized (or distorted) version of $X^{n}$. Consider the error sequence $E_{q}^{n}=X^{n}⊕X_{q}^{n}$, which also models the distortion between $X_{q}^{n}$ and $X^{n}$. The error sequence is shown in [51] (Lemma 11) to resemble a sequence that is distributed according to ${\mathrm{Bernoulli}}^{n}\left(q\right)$ when *n* tends to *∞*.

During reconstruction, a polar decoder of C then observes $Y^{n}$, a noisy version of $X^{n}$ measured through a BSC$\left(p_{A}\right)$. The frozen bits $\bar{V}$ = [V,W] of C are available to the polar decoder in order to estimate *U*^n^ , from which the secret key estimate $\hat{S}$ can be obtained by finding the bit values at indices *j* ∈ {1,2,…,*n*}\F.

Next, a design procedure to implement practical nested polar codes that satisfy these properties is summarised.

Nested polar codes $C\subseteq C_{1}$ must be constructed jointly such that the sets of indices F and $F_{1}$ result in codes that satisfy the security and reliability constraints simultaneously. Suppose the block length *n*, key length $n-m_{1}-m_{2}$, target block-error probability $P_{B}=\mathrm{Pr}\left[S\neq \hat{S}\right]$, and BSC crossover probability $p_{A}$ are given, which depends on the PUF application considered. Then we have the following design procedure [45]:

- Design a polar code C with rate $\frac{n\;-\;m_{1}\;-\;m_{2}}{n}$, corresponding to fixing its indices F that determine the frozen bits. This step is a conventional error-correcting code design task.
- Find the maximum BSC crossover probability $p_{c}$ for which the code C achieves the target block-error probability $P_{B}$, which can be achieved by evaluating the performance of C for a BSC over a crossover probability range. Using the inverse of the star-operation $p_{c}=E\left[q\right]*p_{A}$, the target distortion averaged over a large number of realizations of $X^{n}$ that should be achieved by $C_{1}$ is $E\left[q\right]=\frac{p_{c}-p_{A}}{1-2p_{A}}$. This step can be applied via Monte-Carlo simulations.
- Find an index set $F_{1}$, representing the frozen set of $C_{1}$, such that $F_{1}\subset F$ and the target distortion E[q] is achieved with a minimal amount of helper data. This step can be applied by starting with $F_{1}^{{}^{\prime }}=F$ and then computing the resulting average distortion $E[q^{\prime }]$ obtained from Monte-Carlo simulations. If $E[q^{\prime }]$ is greater than E[q], we remove elements from $F_{1}^{{}^{\prime }}$ according to polarized bit channel reliabilities. This step is repeated until the resulting average distortion $E[q^{\prime }]$ is less than the target (or desired) distortion E[q].

An additional degree of freedom is provided by varying the distortion level in the design procedure above, making the design procedure suitable for numerous applications. Using this degree of freedom, PUFs with different BSC crossover probabilities $p_{A}$ can be supported by using the same nested polar codes with different distortion levels. Similarly, different PUF applications with different target block-error probabilities $P_{B}$ can also be supported by using the same nested codes with different distortion levels.

### 9.2. Designed GS Model Nested Polar Codes

We design nested polar codes to generate a secret key *S* of length $\log|S|\;=\;n\;-\;m_{1}\;-\;m_{2}\;=\;128$ bits, used in the AES. Furthermore, the common target block-error probability for PUFs used in an FPGA is $P_{B}=10^{-6}$ and the common BSC $P_{Y|X}$ crossover probability for SRAM and RO PUFs is $p_{A}=0.15$ [6,36]. We consider these PUF applications and parameters to design nested polar codes that improve on previously proposed codes.

*Code 1*: Suppose a block length of n=1024 bits and a fixed list size of 8 for polar successive cancellation list (SCL) decoders are used for nested codes. First, the code C with rate 128/1024 is designed to determine $p_{c}$, which is defined in the design procedure steps above, obtained by using the SCL decoder. We obtain the crossover probability value $p_{c}=0.1819$, corresponding to a target distortion of E[q]=0.0456. This target distortion is obtained with a minimal helper data *W* length of $m_{2}=650$ bits.

*Code 2*: Suppose a block length of n=2048 bits. Applying the design procedure steps given above, we obtain for Code 2 the value $p_{c}=0.2682$, resulting in a target distortion of E[q]=0.1689. This target distortion is obtained with a minimal helper data *W* length of $m_{2}=611$ bits.

For these nested polar code designs, the error probability $P_{B}$ is considered as the average error probability over a large number of input realizations, corresponding to a large number of PUF circuits that have the same circuit design. This result can be improved by satisfying the target error probability for each input realization, which can be implemented by using the maximum distortion rather than E[q] in the design procedure discussed above. A block-error probability that is ≤$10^{-6}$ can be guaranteed for 99.99% of all realizations of input $X^{n}$ by including an additional 32 bits for the helper data *W* for Code 1 and an additional 33 bits for Code 2. The numbers of additional bits included are small because the distortion *q* has a small variance for the block lengths considered. For code comparisons below, we depict the sizes of helper data needed to guarantee the target block-error probability of $P_{B}=10^{-6}$ for 99.99% of all PUF realizations.

### 9.3. Comparisons of Codes

**Figure 10:**
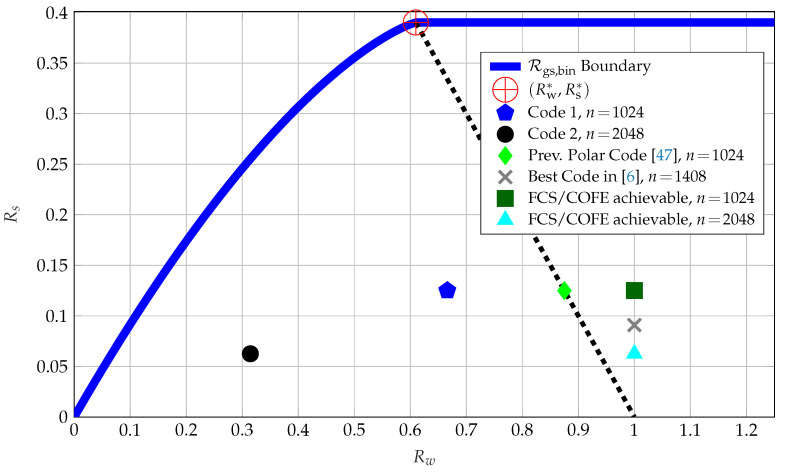
Storage-key rates for the GS model with $p_{A}=0.15$. The $\left(R_{w}^{*},R_{s}^{*}\right)$ point is the best possible point achieved by SW-coding constructions, which lies on the dashed line representing $R_{w}+R_{s}=H\left(X\right)$. The block error probability satisfies $P_{B}\leq 10^{-6}$ and the key length is 128 bits for all code points.

The boundary points of $R_{\mathrm{gs},\mathrm{bin}}$ for $p_{A}=0.15$ are projected onto the storage-key $\left(R_{w},\;R_{s}\right)$ plane and depicted in Figure 10. The point $\left(R_{s}^{*},\;R_{w}^{*}\right)$, defined in Section 3.1, is also depicted. Furthermore, we use the random coding union bound from [44] (Theorem 16) to obtain the rate pairs that can be achieved by using the FCS or COFE. These points are shown in Figure 10 in addition to the rate tuples achieved by the previous SW-coding based polar code design from [47], and Codes 1 and 2 discussed above.

The COFE and FCS result in a storage rate of 1 bit/source-bit, which is strictly suboptimal. The previous SW-coding based polar code construction in [47] achieves a rate tuple such that $R_{s}+R_{w}=1$ bit/source-bit, as expected because it is an SW-coding construction that corresponds to a syndrome coding method in the binary case. The previous SW-coding based polar code construction improves the rate tuples achieved by the COFE and FCS in terms of the ratio of key vs. storage rates. Code 1 achieves the key-leakage-storage tuple of (0.125,0.666,0.666) bits/source-bit and Code 2 of (0.063,0.315,0.315) bits/source-bit, which significantly improve on all previous code constructions without any private key assumption. Thus, Codes 1 and 2 results also suggest that for these parameters increasing the block length increases the $R_{s}/R_{w}$ ratio, which is 0.188 for Code 1 and 0.199 for Code 2. Furthermore, the privacy-leakage and storage rate tuple achieved by Code 2 cannot be achieved by using previous constructions without applying the time sharing method, because Code 2 achieves the privacy-leakage (and storage) rate of 0.315 bits/source-bit that is less than the minimal privacy-leakage (and storage) rates $R_{\ell }^{*}=R_{w}^{*}=H_{b}\left(p_{A}\right)\approx 0.610$ bits/source-bit that can be achieved by using previous code constructions.

To find an upper bound on the the ratio of key vs. storage rates for the maximum secret-key rate point, we apply the sphere packing bound from [52] (Equation (5.8.19)) for the channel $p_{A}=0.15$ and code parameters n=1024, and $P_{B}=10^{-6}$. The sphere packing bound shows that the rate of C, as depicted in Figure 9, must satisfy $R_{C}\leq 0.273$ bits/source-bit. Suppose the key rate is fixed to its maximum value $R_{s}=R_{C}$ and the storage rate is fixed to its minimum value $R_{w}=1-R_{C}$, so we have the ratio of $R_{s}/R_{w}\leq 0.375$. Similarly, for n=2048 we obtain the ratio of $R_{s}/R_{w}\leq 0.437$. The two finite-length results that are valid for WZ-coding constructions with nested codes indicate that ratio of key vs. storage rates achieved by Codes 1 and 2 can be further increased. Using different nested polar codes that improve the minimum-distance properties, as in [53], or using nested algebraic codes for which design methods are available in the literature, as in [54], one can reduce the gaps to the finite-length bounds calculated for nested code constructions. We remark again that such optimality-seeking approaches, for example, based on information-theoretic security, provide the right insights into the best solutions for the digital era’s security and privacy problems.

## 10. Discussions and Open Problems

- We want to use low-complexity scalar quantizers after transformation without extra secrecy leakage; however, the decorrelation efficiency metric does not fully represent the dependency between transform coefficients. What is the right metric to use for choosing the transform used in combination with scalar quantizers? Is mutual information between transform coefficients an appropriate metric for this purpose? The choice of the transform should also depend on a reliability metric such as SNR-packing efficiency so that the transform, quantizers, and the error-correction codes can be designed jointly. What is the right reliability metric for this purpose?
- It is shown in [36] that the ambient temperature and supply voltage affect the RO outputs deterministically rather than adding extra random noise, which was assumed in the RO PUF literature. What are the right output models for common PUF types, i.e., what are the deterministic and random components, and how are they related?
- SRAM PUFs are already used in products. In the literature there is no extensive analysis of the output correlations between different SRAMs in the same device possibly because SRAM outputs are binary and it is difficult to model the correlation between binary symbols. However, SRAM outputs are modeled in [6] as binary-quantized sums of independent Gaussian random variables. Is it possible to determine or approximate the correlations between the Gaussian random variables of different SRAMs? If yes, this might be useful for an attacker to obtain information about the secret sequence generated from the SRAM PUF output, which causes extra secrecy leakage.
- The transform-coding approach discussed above provides reliability guarantees for RO arrays with random outputs, which considers an average over all ROs manufactured. The worst case scenario is when the transform coefficient value is on the quantization boundary, for which the secret-key capacity is 0 bit. If one replaces the average reliability metric used above by a lower bound on the reliability of each RO, i.e., a worst-case scenario metric, how would this change the rate of the error-correction code used? For a fixed code, what should be the optimal bound on the reliability of each RO to maximize the yield, i.e., the percentage of ROs among all manufactured ROs for which the worst-case reliability guarantee is satisfied?
- Are the WZ problem and the GS model operationally equivalent?
- Linear block-code constructions discussed above are for uniformly-distributed PUF outputs. Can one construct other (random) linear block codes that are asymptotically optimal for nonuniform PUF outputs? Is it necessary to use an extension of the COFE for this purpose?
- Consider the nested polar code design procedure given above. Construction of a code for n≤512 is not possible with the procedure discussed above because $q*p_{A}$ increases with increasing *q* for q∈[0,0.5]. Is it possible to construct a nested polar code for n=512 by improving the decoder and the code design procedure?

## Figures and Tables

**Table 1:** Average decorrelation-efficiency results for RO outputs.

	DWHT	DCT	DHT
$\eta_{c}$ for 16×16	0.9988	0.9987	0.9986
$\eta_{c}$ for 8×8	0.9977	0.9978	0.9978

**Table 2:** Code-design constraints.

$C_{\max}$	20	19	18	17	16
${\bar{P}}_{c}$	0.9844	0.9860	0.9875	0.9889	0.9902
$K_{\max}$	3	3	3	3	3
n	259	255	250	224	144
e	25	23	21	20	18

## Data Availability

Not applicable.

## References

[1] Shannon C.E. (1949). Communication theory of secrecy systems. Bell Syst. Tech. J..

[2] Kahn D. (1967). The Codebreakers: The Story of Secret Writing.

[3] Schneier B. (1996). Applied Cryptography: Protocols, Algorithms, and Source Code in C.

[4] Böhm C., Hofer M. (2012). Physical Unclonable Functions in Theory and Practice.

[5] Gassend B., Clarke D., Dijk M.V., Devadas S. Silicon physical random functions. Proceedings of the 9th ACM Conference on Computer and Communications Security.

[6] Maes R., Tuyls P., Verbauwhede I. A Soft Decision Helper Data Algorithm for SRAM PUFs. Proceedings of the 2009 IEEE International Symposium on Information Theory.

[7] Goldreich O. (1998). Modern Cryptography, Probabilistic Proofs and Pseudorandomness.

[8] Pappu R. (2001). Physical One-Way Functions. Ph.D. Thesis.

[9] Wyner A.D. (1975). The wire-tap channel. Bell Syst. Tech. J..

[10] Palanca A., Evenchick E., Maggi F., Zanero S. (2017). A stealth, selective, link-layer denial-of-service attack against automotive networks. Detection of Intrusions and Malware, and Vulnerability Assessment.

[11] Lee Y.S., Lee H.J., Alasaarela E. Mutual authentication in wireless body sensor networks (WBSN) based on Physical Unclonable Function (PUF). Proceedings of the 2013 9th International Wireless Communications and Mobile Computing Conference (IWCMC).

[12] Simpson E., Schaumont P. (2006). Offline hardware/software authentication for reconfigurable platforms. International Workshop on Cryptographic Hardware and Embedded Systems.

[13] Herder C., Yu M., Koushanfar F., Devadas S. (2014). Physical Unclonable Functions and Applications: A Tutorial. Proc. IEEE.

[14] Huth C., Guillaume R., Strohm T., Duplys P., Samuel I.A., Güneysu T. (2016). Information reconciliation schemes in physical-layer security: A survey. Comput. Netw..

[15] Lim D., Lee J.W., Gassend B., Suh G.E., Dijk M.V., Devadas S. (2005). Extracting Secret Keys From Integrated Circuits. IEEE Trans. Very Large Scale Integr. Syst..

[16] Mandal M.K., Sarkar B.C. (2010). Ring oscillators: Characteristics and Applications. Indian J. Pure Appl. Phys..

[17] Suh G.E., Devadas S. Physical Unclonable Functions for Device Authentication and Secret Key Generation. Proceedings of the 2007 44th ACM/IEEE Design Automation Conference.

[18] Guajardo J., Kumar S.S., Schrijen G.J., Tuyls P. (2007). FPGA Intrinsic PUFs and Their Use for IP Protection. International Workshop on Cryptographic Hardware and Embedded Systems.

[19] Günlü O., Kramer G., Skorski M. Privacy and secrecy with multiple measurements of physical and biometric identifiers. Proceedings of the 2015 IEEE Conference on Communications and Network Security (CNS).

[20] Dodis Y., Ostrovsky R., Reyzin L., Smith A. (2008). Fuzzy extractors: How to generate strong keys from biometrics and other noisy data. SIAM J. Comput..

[21] Juels A., Wattenberg M. A fuzzy commitment scheme. Proceedings of the 6th ACM Conference on Computer and Communications Security.

[22] Ignatenko T., Willems F.M.J. (2009). Biometric systems: Privacy and secrecy aspects. IEEE Trans. Inf. Forensics Secur..

[23] Günlü O., Kramer G. (2018). Privacy, Secrecy, and Storage With Multiple Noisy Measurements of Identifiers. IEEE Trans. Inf. Forensics Secur..

[24] Günlü O., İşcan O. DCT Based Ring Oscillator Physical Unclonable Functions. Proceedings of the 2014 IEEE International Conference on Acoustics, Speech and Signal Processing (ICASSP).

[25] Maiti A., Schaumont P. (2011). Improved ring oscillator PUF: An FPGA-friendly secure primitive. J. Cryptol..

[26] Ahlswede R., Csiszár I. (1993). Common Randomness in Information Theory and Cryptography—Part I: Secret Sharing. IEEE Trans. Inf. Theory.

[27] Maurer U.M. (1993). Secret Key Agreement by Public Discussion from Common Information. IEEE Trans. Inf. Theory.

[28] Günlü O., Schaefer R.F., Poor H.V. (2020). Biometric and physical identifiers with correlated noise for controllable private authentication. arXiv.

[29] Günlü O., Schaefer R.F., Kramer G. Private authentication with physical identifiers through broadcast channel measurements. Proceedings of the 2019 IEEE Information Theory Workshop (ITW).

[30] Ignatenko T., Willems F.M. (2010). Information leakage in fuzzy commitment schemes. IEEE Trans. Inf. Forensics Secur..

[31] Cover T.M., Thomas J.A. (2012). Elements of Information Theory.

[32] Günlü O., Kernetzky T., İşcan O., Sidorenko V., Kramer G., Schaefer R.F. (2018). Secure and Reliable Key Agreement with Physical Unclonable Functions. Entropy.

[33] Maiti A., Casarona J., McHale L., Schaumont P. A Large Scale Characterization of RO-PUF. Proceedings of the 2010 IEEE International Symposium on Hardware-Oriented Security and Trust (HOST).

[34] Schwarz G. (1978). Estimating the dimension of a model. Ann. Stat..

[35] Sugiura N. (1978). Further analysis of the data by Akaike’s information criterion and the finite corrections. Commun. Stat. Theory Methods.

[36] Günlü O., İşcan O., Kramer G. Reliable secret key generation from physical unclonable functions under varying environmental conditions. Proceedings of the 2015 IEEE International Workshop on Information Forensics and Security (WIFS).

[37] Lin S., Costello D.J. (2004). Error Control Coding.

[38] Ohm J.R. (2015). Multimedia Signal Coding and Transmission.

[39] Wang R. (2012). Introduction to Orthogonal Transforms: With Applications in Data Processing and Analysis.

[40] Günlü O., Schaefer R.F. Low-Complexity and Reliable Transforms for Physical Unclonable Functions. Proceedings of the 2020 IEEE International Conference on Acoustics, Speech and Signal Processing (ICASSP).

[41] Maes R., Herrewege A.V., Verbauwhede I. (2012). PUFKY: A fully functional PUF-based cryptographic key generator. Cryptographic Hardware Embedded Systems.

[42] Rukhin A., Soto J., Nechvatal J., Smid M., Barker E. (2001). A Statistical Test Suite for Random and Pseudorandom Number Generators for Cryptographic Applications.

[43] Hong Y. (2011). On Computing the Distribution Function for the Sum of Independent and Nonidentical Random Indicators.

[44] Polyanskiy Y., Poor H.V., Verdú S. (2010). Channel Coding Rate in the Finite Blocklength Regime. IEEE Trans. Inf. Theory.

[45] Günlü O., İşcan O., Sidorenko V., Kramer G. (2019). Code Constructions for Physical Unclonable Functions and Biometric Secrecy Systems. IEEE Trans. Inf. Forensics Secur..

[46] Wyner A.D., Ziv J. (1976). The rate-distortion function for source coding with side information at the decoder. IEEE Trans. Inf. Theory.

[47] Chen B., Ignatenko T., Willems F.M., Maes R., van der Sluis E., Selimis G. A Robust SRAM-PUF Key Generation Scheme Based on Polar Codes. Proceedings of the GLOBECOM 2017—2017 IEEE Global Communications Conference.

[48] Slepian D., Wolf J. (1973). Noiseless coding of correlated information sources. IEEE Trans. Inf. Theory.

[49] Maurer U., Wolf S. Information-theoretic key agreement: From weak to strong secrecy for free. Proceedings of the International Conference on the Theory and Applications of Cryptographic Techniques.

[50] Arikan E. (2009). Channel Polarization: A Method for Constructing Capacity-Achieving Codes for Symmetric Binary-Input Memoryless Channels. IEEE Trans. Inf. Theory.

[51] Korada S.B., Urbanke R.L. (2010). Polar Codes are Optimal for Lossy Source Coding. IEEE Trans. Inf. Theory.

[52] Gallager R.G. (1963). Low-Density Parity-Check Codes.

[53] Günlü O., Trifonov P., Kim M., Schaefer R.F., Sidorenko V. (2020). Randomized Nested Polar Subcode Constructions for Privacy, Secrecy, and Storage. arXiv.

[54] Jerkovits T., Günlü O., Sidorenko V., Kramer G. (2020). Nested Tailbiting Convolutional Codes for Secrecy, Privacy, and Storage. arXiv.

